# A survey of water chemistry used in zebrafish facilities and their effects on early zebrafish development

**DOI:** 10.12688/f1000research.134520.2

**Published:** 2024-08-20

**Authors:** Cosima S. Porteus, Ella Waples, Anna Dempsey, Gregory Paull, Rod W. Wilson

**Affiliations:** 1 Biological Sciences, University of Toronto Scarborough, Toronto, Ontario, M1C 1A4, Canada; 2 Biosciences, University of Exeter, Exeter, Devon, EX4 4QD, UK

**Keywords:** Growth, development, zebrafish, water chemistry, pH, temperature, conductivity, salinity, polyculture

## Abstract

**Background:**

There are a variety of published standard methods and water chemistry recommendations for zebrafish ( *Danio rerio*) husbandry, but empirical evidence for their justification is often lacking, as is information on some variables that have important biological effects on fish. Importantly, these different recommendations could contribute to variability in results and fish welfare between or within institutions.

**Methods:**

Here we document the current range of water chemistry used by various research institutions around the world and report initial findings on their effects on the development and growth of zebrafish. Over 40 institutes responded to a survey that revealed a large variation in water chemistry used for zebrafish husbandry including differences in the set-points and acceptable ranges for temperature, pH and conductivity. In subsequent experiments, zebrafish ( *D. rerio*, WIK) embryos/larvae exposed to a large range of salt concentrations (50μM to 10mM Na ^+^ or 30 – 2500 μS/cm) and CO _2_ levels (400 – 8,000 μatm).

**Results:**

Larvae exposed to the lowest salt concentration (5 μM Na ^+^ or < 30μS/cm) had a slower response to touch and their swim bladders were not inflated. Larvae exposed to 5-100 μM Na ^+^ were 5 % shorter in total body length than those exposed to higher salt concentrations (>100 μM Na ^+^). Zebrafish embryo/larvae exposed to intermediate pCO _2_ values (~2000 μatm) were 1 to 3.5% longer than those exposed to either ambient (400 μatm) or higher (4000 μatm) pCO _2_, but pCO _2_ did not affect developmental endpoints up to 4 dpf.

**Conclusions:**

Overall, we highlight the magnitude of variation in water chemistry used within zebrafish research and provide some empirical evidence to show that not all of these water conditions might be optimal for developing zebrafish and reproducibility of research, although further research is necessary to determine longer-term effects of water chemistry on older larvae, juveniles and adults.

## Introduction

Fish physiologists have known for decades that ion levels have a dramatic influence on the physiology and health of fish generally, but also specifically on the ability to cope with ion and acid-base disturbances ( Brauner *et al.,* 2019). Sodium (Na ^+^) and chloride (Cl ^-^) are the two major ions that freshwater fish must actively take up from the water for survival. Critically, they are also the key counter-exchange ions that freshwater fish require for regulating their internal acid-base balance (i.e. for excreting excess acid or base, respectively). These are important for internal pH homeostasis which in turn is vital for ensuring all cellular proteins maintain functionality. It is well established that animals that are kept under suboptimal conditions must devote more energy to maintaining homeostasis, rather than using it for growth, gamete production and immune function ( Wootton, 1999). For example, rainbow trout ( *Oncorhynchus mykiss*) acclimated to low NaCl concentrations had double the blood acidosis after exercise and took twice as long to recover compared to fish acclimated to very high NaCl ( Tang *et al.,* 1989). Similarly, rainbow trout exposed to elevated CO _2_ recovered their blood pH quicker when either NaCl, or Ca ^2+^, or HCO _3_^-^ were higher in the water ( Iwama and Heisler, 1991; Larsen and Jensen, 1997). Importantly, fish maintained at the lower ion concentrations in these same studies were often incapable of restoring blood pH at all, and the ranges tested (approximately 0.05 to 5 mM) encompass the range typically found in natural freshwater environments, and in zebrafish facilities.

The success of the zebrafish as a model species is not surprising given their high fecundity, transparent embryos, short life cycle as well as their natural distribution within the floodplains of the Ganges and Brahmaputra Rivers, which vary greatly in water chemistry ( Lee *et al.*, 2020). This natural history accounts for much of the tolerance of zebrafish to a wide range of physiological stressors. However, despite the ability of zebrafish to survive wide-ranging water chemistry parameters, certain conditions may not be optimal for thriving. Currently, around 87% of zebrafish used for scientific research are kept in recirculating systems ( Lawrence, 2007; Lidster *et al.*, 2017). CO _2_ can accumulate in these systems unless significant CO _2_-stripping effort is employed to remove it, i.e. more than just sufficient aeration to restore normal oxygen levels ( Hu *et al.*, 2011; Summerfelt *et al.,* 2000). This is due to CO _2_ having a 30 times greater solubility in water compared to O _2_, and also the slower reaction speed of CO _2_ hydration and its reversal. Therefore, it is more difficult to remove the respiratory CO _2_ excreted by fish than it is to replace the O _2_ they consume and, as a result, CO _2_ gradually accumulates on each pass through the system whilst normal O _2_ levels are maintained ( Hu *et al.*, 2011). It has been previously established that zebrafish can cope with high pCO _2_ (partial pressure of CO _2_) conditions, if kept in hard water with very high levels of NaCl (13 mM) and HCO _3_^-^ (5 mM) ( Mölich and Heisler, 2005). However, just because zebrafish can tolerate these pCO _2s_, they might not be in a good physiological state. Therefore, despite the ability to regulate blood acid-base balance, living in high CO _2_ water has further, more holistic impacts on fish performance. For example, it reduces growth and efficiency of digestion in juvenile and adult Atlantic salmon ( *Salmo salar*; Fivelstad, 2013; Mota *et al.,* 2020) and adult Atlantic cod ( *Gadus morhua*; Tirsgaard *et al.,* 2015) and reduces the conversion of yolk into growth in larval pink salmon ( *Oncorhynchus gorbuscha*; Ou *et al.,* 2015). Also, 12 weeks exposure to elevated CO _2_ in Atlantic salmon caused 72 differentially expressed genes, 60 being down-regulated with a predominance of those being associated with immune responses ( Mota *et al.,* 2020). However, the are no studies on zebrafish that have assessed the impact of pCO _2_ or its interaction with NaCl.

A surprisingly wide range of water chemistry is used throughout the zebrafish community ( Lawrence, 2007). For example, Na ^+^ concentration varies more than 350-fold in various “recipes” for freshwater media established for use in raising larvae until the first feeding stage (4-5 dpf, Table 1). Moreover, these variations not only exist between facilities but even within them, with different researchers having different preferred larval media within the same institution. Variations also exist between different life stages within a research group and between animals destined for stock or experiments. By discussing this with colleagues and reading what has been reported elsewhere ( Lee *et al.,* 2022), we found that this is due to differences in standards for the particular field or due to historical use (i.e. how researchers were trained to do it). For example, the standard solution used for toxicological and pharmaceutical testing in Europe is ISO 7346-3 ( ISO7346-3, 1996) which states that this solution can be diluted up to 6 times to reduce water hardness in order to minimize the effects of salts on the bioavailability and uptake of toxicant chemicals (including pharmaceuticals) being studied ( Pinheiro *et al.*, 2021). At the same time, fish destined for maintaining stock in the same facility might be raised by facility staff in media recommended in The Zebrafish Book ( Westerfield, 2007). Furthermore, posts on online chat groups indicate that researchers change the original recipes of standard solutions such as E2 or E3 ( Nüsslein-Volhard and Dahm, 2002), introducing even more variability and uncertainty in methods used and results. Finally, it is not clear what empirical evidence these recommended “recipes” for freshwater media are based upon regarding their impact on either fish health or research outcomes.

**Table 1.** Ionic composition (mM) of commonly used artificial freshwater media for zebrafish embryos and larvae (0-22 dpf) reported in the literature. This clearly shows the large fold difference (between the lowest and highest ion concentrations of all media, far right column) between the main ion concentrations between these different solutions.

Salt		Artificial Salts-Based Media				Marine Salts		Fold Difference
(mM)	OECD ^1^	ISO ^2^	E3 ^3^	E2 ^3^	Danieau’s Solution ^4^	0.06 ppt ^5^	2.0 ppt ^6^	
**[Na** ^**+**^ **]**	0.77	0.15	4.96	7.90	58.00	0.8	26.8	**387**
**[Cl** ^**-**^ **]**	4.08	0.82	6.60	8.75	58.00	0.93	31.2	**71**
**[Ca** ^**2+**^ **]**	2.00	0.40	0.33	0.50	0.60	0.018	0.59	**111**
**[HCO**_**3**_^**-**^ **]**	0.77	0.15	0	0.35	0	0.004	0.11	**[0-1.4]**
**[Mg** ^**2+**^ **]**	0.50	0.10	0.40	0.50	0.40	0.091	3.02	**33**
**Conductivity (μS/cm)**	358	162	817	1169	6611	118	3796	**56**
**Salinity (ppt)**	**0.32**	**0.08**	**0.40**	**0.58**	**3.61**	**0.06**	**2.00**	**60**

^1^ OECD Test No. 236: FET, 2013.

^2^ ISO, 1996.

^3^ Nüsslein-Volhard and Dahm, 2002.

^4^ Gustafson *et al.*, 2012.

^5^ Westerfield, 2007.

^6^ Sawant *et al.*, 2001.

In addition to the immediate effects of differences in water chemistry, we propose that raising zebrafish under such varied conditions can also affect their response to environmental variables in later life as previously shown in juvenile European sea bass ( *Dicentrarchus labrax*; Fokos *et al.*, 2017). Developmental plasticity has been shown for stressors such as temperature ( Schaefer and Ryan, 2006) and hypoxia ( Robertson *et al.,* 2014) and exposure during early life to high CO _2_ can alter the ventilatory sensitivity of zebrafish in adulthood ( Vulesevic and Perry, 2006). Therefore, early exposures to different water chemistry conditions could affect how zebrafish respond to various stressors later in life, and potentially give rise to a range of phenotypes when exposed to various stressors or toxicants as juveniles or adults. This in turn causes discrepancy between the outcomes of similar studies conducted at different institutions. Indeed, methodological issues stemming from insufficient understanding of factors that may influence experimental results has been previously identified as a factor that can contribute to the reproducibility and replicability of results in zebrafish research studies ( Gerlai, 2019). In principle, poor reproducibility and replicability is likely to result in a greater total number of animals being used in research, as it may encourage the repeat of experiments to explore what the most accurate or representative outcome of research actually is.

The purpose of the current study was to gather quantitative data regarding the variation in water chemistry used by different institutions using zebrafish and to explore how this may affect the early development of zebrafish. To achieve this, we then measured standard developmental endpoints ( Kimmel *et al.,* 1995) and the growth of zebrafish under different water chemistry regimes up to the independent feeding stage. We hypothesized that the growth and development of zebrafish would be affected by changes in salt level and pCO _2_ and that these differences would be more pronounced in high and low salt extremes and at the highest CO _2_ level.

## Methods

### Survey info

A survey to determine the water chemistry ranges used to house zebrafish was sent to the global zebrafish community (distributed to the Zebrafish Husbandry Association and the British Zebrafish Husbandry Association list servers, and other institutions on an individual basis) between February 2018 and August 2019 (University of Exeter Ethics Committee, ID 5542334). Forty institutions/zebrafish facilities in 12 countries from Europe, USA, Canada, Australia, New Zealand and Asia responded to the survey. We obtained informed consent from all the participants in writing to share these data without disclosing institutional information. The survey questions focused on identifying the target and actual operating range of water chemistries known to have effects on fish (see Supplementary Materials for the Survey). Specifically, the questionnaire requested detailed information regarding source water, salt addition (type, manufacturer and concentrations/ratios used), water filtration methods, water chemistry parameters monitored, and operating conditions for the important life stages of zebrafish development, i.e. embryo, larvae and adult. In the questionnaire, the institutions were also requested to define the age ranges for the different stages of development as this varied between institutions. Sodium is the dominant cation across global freshwater ecosystems ( Pinheiro *et al.,* 2021) as well as in most established zebrafish media. However, given that the freshwater media commonly used to culture zebrafish are synthetic combinations of many individual salts, not just those containing sodium, for simplicity throughout we refer to the different treatments in terms of their total sodium concentration. Total salt concentrations were calculated using the target conductivity (which is proportional to the sum of all ions present) or the midpoint between the reported minimum and maximum conductivity for those institutions where a target conductivity was not provided. Where minimum and maximum values were given, these refer to the point at which action would be taken at the relevant local institute to correct the water chemistry. Throughout the manuscript, system water refers to the standard water used for housing fish in each facility, but the salt concentration in these systems is typically different at each site. For example, the sodium concentration of system water in these facilities varies from 2.1 mM to 10.6 mM with most between 3.2 and 6.3 mM.

### Water samples for CO _2_ determination from 3 UK zebrafish facilities

Water samples were collected from three prominent zebrafish facilities in the UK. The rationale for choosing the three facilities was that they represented typical mid-to-large scale zebrafish facilities; they used commercially available tanks and recirculating filtration systems to house their fish; they were well established with standardised husbandry methods and experienced animal care staff and management; and they were well stocked with fish at densities typically reported for zebrafish facilities (4-10 fish/L) and within set guidelines (reviewed by Lee *et al.*, 2022). All of these factors suggested that the water chemistry ranges recorded by these institutions would be a good representation of other facilities. System water was sampled from 3 to 4 different locations within each facility (10 samples in total). Water samples (12 ml) were preserved in gas-tight vials with mercuric chloride (HgCl _2_) according to standard methods ( Dickson *et al.,* 2007) and stored at 4°C. The samples were transported to the University of Exeter, where salinity and pH _NBS_ were measured upon arrival using a salinity and conductivity system (YSI Model 30, YSI incorporated, Yellow Springs, Ohio, USA), pH meter (Model HI 8314, Hanna Instruments, Leighton Buzzard, UK) and pH probe (Model pHC2401, Radiometer Analytical, Lyon, France) calibrated with National Bureau of Standards (NBS) buffers. System water samples were analysed for dissolved inorganic carbon (DIC) using a custom built system ( Lewis *et al.,* 2007). Measured temperature, salinity, pH and DIC values were used to calculate average water pCO _2_ and total alkalinity (TA – a measure of the water’s acid-neutralizing capacity) using the CO2SYS software using the GEOSECS constants.

### Ethics statement

All experiments were carried out in the University of Exeter Aquatic Resources Centre (ARC), and procedures were approved by the Home Office (License No P88687E07).

### Experimental animals

Zebrafish were bred from the Wild Indian Karyotype (WIK) strain, by small group batch spawning, consisting of 4 individuals per sex. The fish were placed in the breeding tank the previous night and spawning was initiated from 08:55 with lights turning on gradually, and embryos were collected at 09:45. The standard breeding conditions at the University of Exeter are 28±1°C, using system water with an average pH of 7.5. At the time of this study, the system water was reverse osmosis (RO) reconstituted with 2.1 mM calcium chloride dihydrate (CaCl _2_•2H _2_O), 0.53 mM magnesium sulphate heptahydrate (MgSO _4_•7H _2_O), 0.39 mM sodium bicarbonate (NaHCO _3_), 0.08 mM potassium chloride (KCl), and 0.5 mM sodium chloride (NaCl).

### Experimental design

All egg test procedures were carried out following the guidelines for the OCED FET Test No. 236 ( OECD, 2013), using larvae at 0 to 4 days post fertilisation (dpf) as follows. Embryos were separated into groups of 24 individuals per treatment as specified in the FET assay ( OECD, 2013) and transferred <90 minutes post fertilisation into group treatment conditions into a 6 well plate in the CO _2_ incubator, via a pipette (15 embryos per 10 ml treatment solution), during the 4 to 16 cell cleavage stage ( OECD, 2013), in a 28°C environment. Fertilisation success was checked and recorded 3 to 4 hours post fertilisation (hpf) ( Brannen *et al.*, 2010). For test results to be valid a fertilisation success >70% must be achieved, in accordance with the OECD FET assay guidelines ( OECD, 2013). The first twelve fertilised embryos were chosen per treatment and transferred using a 200 μl commercially available pipette with a widened tip opening into individual wells of a 12-well plates containing 1 ml of the same initial treatment solution ( OECD, 2013). This resulted in each embryo being kept in 1.2 ml at 28±1°C in the respective CO _2_ incubator by 4 hpf. All the experiments were performed on two different batches (experimental unit) of embryos for a total sample size of 19-24 embryos per treatment, except Experiment 1, 2000 μtm at 5 μM Na ^+^ where N=11 because this lowest salt level was omitted in the first batch of embryos used. A total of 192 total animals used for experiment 1 and 336 for experiment 2 (Supplementary tables S1 and S2). The only reason to exclude an animal from an experiment was natural mortality or a body shape score of less than 5 (mildly to severely deformed). A body shape less than 5, absence of a heart beat and pericardial oedema were considered a humane endpoint and fish were humanely killed once this was noted and confirmed (about 0.5% of larvae).

Embryos/larvae were removed from the CO _2_ incubator daily (between 09:30 and 10:30, for <15 minutes) and checked for survival and stages of development ( Kimmel *et al.,* 1995): tail detachment and somite formation at 24 hpf, heartbeat and hatching success at 48 hpf, and response to touch and body shape scored between 1-5 ( Brannen *et al.,* 2010) at 72 and 96 hpf. Survival rate >80% is required in system water to allow for validity of test results, as per the OCED FET assay No. 236 guidelines ( OECD, 2013). Salt solutions were kept at 28±1°C and were bubbled every morning for at least 30 minutes to reach the desired CO _2_, then 1 ml of water from the well was replaced with fresh bubbled solution and the well plate returned to the CO _2_ incubator and the order was switched around to ensure the same plate was not always in the same spot in the incubator.

At 98 hpf larvae were anesthetised using Tricaine (MS-222 4 g/L) until movement stopped (~5 minutes). They were imaged on a Nikon SMZ1500 bright-field microscope at 2X magnification and total lengths were measured for larvae with body shape 5, using Fiji (ImageJ) software ( Schindelin *et al.,* 2012), from the tip of the head to the end of the caudal fin. Larvae order were randomised prior to imaging using the random number generator function in Excel. The researchers were blind to treatment allocation during imaging and length measurement, but not during daily checking of developmental milestones.

### Water chemistry treatments

*Experiment 1: Analar grade salts and CO _2_*

A single stock solution was prepared weekly using 5 mM NaCl, 5 mM CaCl _2_•2H _2_O, 1.8 mM MgSO _4_•7H _2_O, 0.5 mM potassium sulphate (K _2_SO _4_), and 5 mM NaHCO _3_ in ultrapure (MilliQ) water by adding each salt one at a time and completely dissolving it before adding the next one. The stock solution was then diluted to achieve 250 ml of the following nominal sodium concentrations of 5, 50, 100, 200, 500, 1,000, 2,000, 5,000 and 10,000 μM every other day (to ensure less than 20% deviation from nominal values, OECD, 2013) corresponding to the treatments of this experiment. Clean “system water” from the Aquatic Resources Centre (ARC) was used as an additional control. Each solution was gassed to one of the four respective experimental partial pressures of CO _2_ (pCO _2_) and maintained at 28±1°C in a temperature-controlled room. The pCO _2_ measured in this study will be referred to generally as CO _2_ hereafter.

The nominal CO _2_s were 400, 2000, 4000, and 8000 μatm (0.54, 2.75, 5.5 and 11.0 mg l ^−1^, respectively), achieved using AALBORG Mass flow Controllers (CACHE instrumentation, UK), flow range 0–200 ml min ^−1^ for CO _2_ and 0–10 L min ^−1^ for air flow from an air pump (MEDO LA-45B). These gas combinations flowed through a CO _2_ incubator with a sealed lid. Embryos/larvae under treatment were kept in 1ml of solution in 12-well plates, within the gas-tight incubator. The solution was bubbled for 15-30 minutes prior to exposure to allow levels to stabilise with the respective CO _2_ flow. Preliminary experiments showed this method kept consistent CO _2_ levels within the solution.

*Experiment 2: Commercial marine salts and CO _2_*

Stock solutions of a commercial marine salt at a salinity of 35 parts per thousand (ppt) were prepared weekly by dissolving either Instant Ocean or Tropic Marin commercial salts in ultrapure (MilliQ) water. Stock solutions were diluted twice a week to achieve 6 different “freshwater” salinities of 0.08, 0.16, 0.32, 0.64, 1.2 and 2.0 ppt equivalent to sodium concentrations of 1,250, 2,500, 5,000, 10,000, and 20,000, and 30,000 μM. In this experiment E2 and E3 media ( Nüsslein-Volhard and Dahm, 2002) were used for additional comparison. Each solution was gassed to one of three different partial pressures of CO _2_ (pCO _2_) of 400, 2000, and 4000 μatm (0.54, 2.75, and 5.5 mg l ^−1^, respectively) as described above and maintained at 28±1°C.

### Water Chemistry Analysis

Temperature (Model HI 8424 Hanna Instruments, Leighton Buzzard, UK) and pH meter (Model HI 8314, Hanna Instruments, Leighton Buzzard, UK, calibrated with HACH buffers) with a red rod pH electrode (HACH, PHC705) in the individual salt solutions, were measured twice a week. Water samples (12 ml) were collected and preserved as described above. The pH and temperature were measured twice weekly in the well water, by retaining the 1 ml waste collected from each treatment and pooled to obtain a sufficient volume for measurement. Levels of CO _2_ were calculated as described above. Separate water samples were also taken for later analysis of ion concentrations using ion chromatography (Dionex ICS-1000 and ICS-1100).

### Statistical analysis

Statistical analysis on the effect of salt concentration and CO _2_ and their interaction on zebrafish larvae length, was analysed using the PERMANOVA+ ( Anderson, 2008) add on in PRIMER 6.1 ( Clark and Gorley, 2006), but can be performed using R. Data were tested to ensure homogeneity of variance, and a similarity matrix was constructed using Euclidean distance. P-values were calculated using 9999 permutations, and pair-wise comparisons were made to see differences between CO _2_ and salt concentration factors.

## Results

### Survey

**Figure 1.**
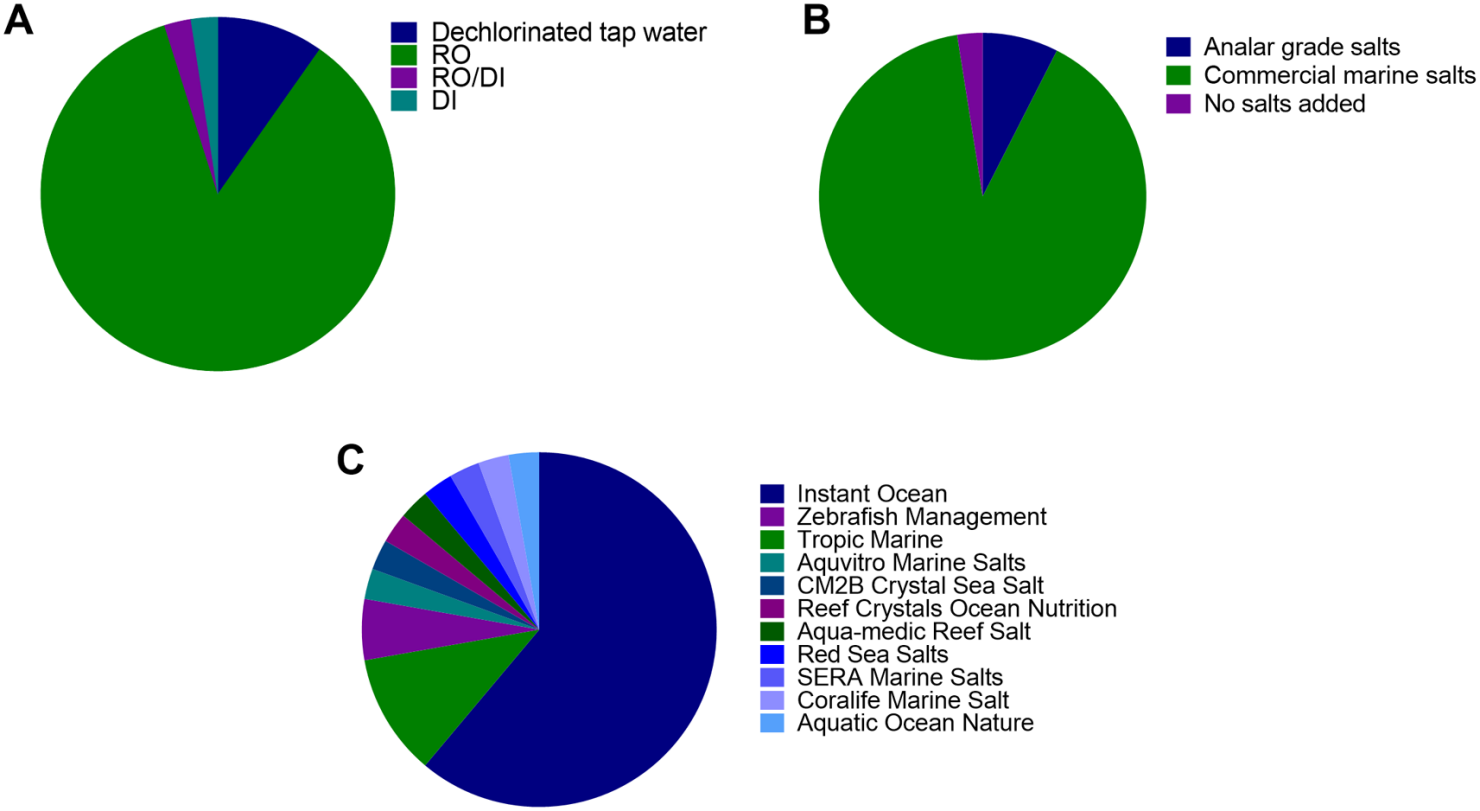
Breakdown of water composition of system water used in 40 zebrafish facilities across the world. Type of make up water used; B) Type of salts used to make up system water; C) Type of commercial salts used to make system water for facilities that use commercial marine salts (most surveyed). RO = reverse osmosis, DI = deionized water.

Of the 40 institutions surveyed, 35 institutes used pure water derived from reverse osmosis (RO), 1 used deionised water (DI), 1 used a combination of reverse osmosis and/or deionised water (RO/DI) and 4 used dechlorinated water ( Figure 1A). To this water, either commercial marine salts (36/40 Institutes) or Analar grade salts (3 institutes) were added to create “system water” ( Figure 1B). However, one institute used dechlorinated local water without any salts added. Eleven varieties of the commercial marine salts were used, with the most common being ‘Instant Ocean’ (22 institutes) followed by Tropic Marin Centre reef salts (TMC 4 institutes) ( Figure 1C).

**Figure 2.**
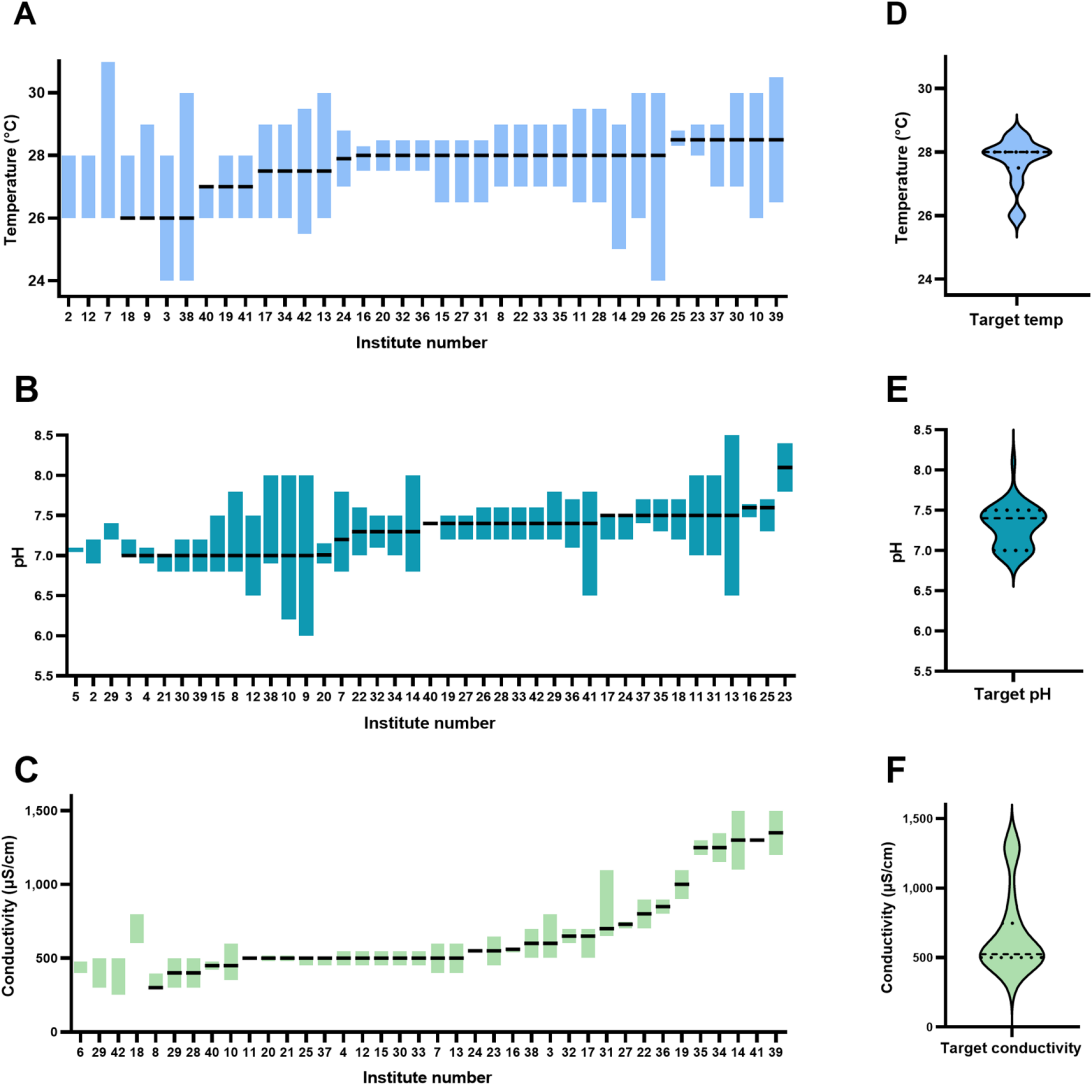
Target and reported range (minimum and maximum) temperature (A), pH (B), and conductivity (C) of the 40 institutes surveyed and violin plots of the target temperature (D), pH (E), and conductivity (F). Note: the data is ordered in terms of target water parameter (temperature, pH, or conductivity) followed by range, therefore x-axis (order of institutes) is different between panels. Dashed lines in the violin plots represent median values, and the dotted lines represent quartiles (in D the median and upper quartiles are the same).

The target temperature of the facilities surveyed varied from 26 to 28.5°C, with a mean of 27.7±2.6°C ( Figure 2). The minimum acceptable temperature was 24°C and the maximum was 31°C among all institutes surveyed. For a chosen target temperature, the minimum range was 0.8°C and the maximum range was 6°C. The target pH varied between 7.0 and 8.1, with a mean of 7.3±0.6 ( Figure 2). The minimum acceptable pH reported was 6.0 and the maximum was 8.4 among all institutes surveyed. The acceptable pH range varied from 0 to 1.8 pH units within an institute. The target conductivity of the facilities surveyed varied from 300 μS/cm to 1350 μS/cm (equivalent to salinities of 0.13 – 0.65 ppt), with a mean of 667 ± 156 μS/cm (0.30 ppt). The minimum acceptable conductivity was 300 and the maximum was 1500 μS/cm (0.13 and 0.73 ppt, respectively) among all institutes surveyed. The acceptable conductivity range (deviation from mean) varied from 30 μS/cm to 450 μS/cm within an institute. Based on the reported target conductivity, we estimated the sodium concentrations of the system water of all the institutions surveyed. This suggests a minimum Na ^+^ concentration of 0.65 mM and a maximum of 11 mM, with an average of 4.95 mM. Of all the institutions surveyed only one routinely measured pCO _2_.

The most common freshwater media used for raising zebrafish up to 5 dpf (independent feeding) was E3 (4.96 mM Na ^+^), followed by system water (which was rarely the same composition between institutes and ranged from 3.2 to 11 mM Na ^+^) and egg water (0.8 mM Na ^+^ or 0.06 ppt; Westerfield, 2007). Most facilities (26 institutions) grew the larvae in system water in static tanks between 5 dpf and 14 dpf, and these were transferred to a recirculating system using system water between 14 and 22 dpf. The second most common practice (at 5 out of 40 institutions) for raising zebrafish larvae between 5-14 dpf was a polyculture system, growing these together with marine rotifers and algae at salinities of 2 to 7 ppt. Based on the salinities used we estimate that the sodium concentrations varied by 75-fold between institutions between 0 and 5 dpf, by 122-fold between 5 and 14 dpf, and by 17-fold between 14 and 22 dpf. The largest variation in sodium concentration within an institution was 86-fold between eggs and 5-14 dpf larvae. In one institution, based on the transfer of 14 dpf larvae from 7 ppt to a recirculating system using system water, we estimated that these larvae would experience a 20.5-fold change in sodium concentration over a course of approximately 24 hours.

### Water CO _2_ measured from 3 UK zebrafish facilities

The water samples taken from the 3 major zebrafish facilities in the UK revealed considerable variability in their carbonate chemistry ( Table 2). These were compared to water samples from our own facility at Exeter for reference ( Table 3). The pH in the 3 major UK facilities was between 6.29 and 7.20, with a mean of 7.12. The pCO _2_ varied between 1,468 and 2,826 μatm, with an average of 1,984±431 μatm. The alkalinity varied between 53 and 613 μM, with an average of 372 μM. Our facility, which had gone through a recent expansion and therefore had low fish densities by comparison to those sampled had average values of 7.0, 465 μatm, and 253 μM for pH, pCO _2_ and alkalinity.

**Table 2.** Water chemistry parameters measured in 3 major UK zebrafish facilities at multiple sites in those facilities (samples 1-4).

Anonymized UK Zebrafish Facilities	pCO _2_ (μatm)	pH	Alkalinity (μM)
**ZF Facility 1 - Sample #1**	2,826	6.97	469.3
**ZF Facility 1 - Sample #2**	1,840	7.12	431.8
**ZF Facility 1 - Sample #3**	1,668	7.20	470.9
**ZF Facility 2 - Sample #1**	2,150	7.13	515.9
**ZF Facility 2 - Sample #2**	1,468	7.10	329.5
**ZF Facility 2 - Sample #3**	2,553	7.13	612.8
**ZF Facility 2 - Sample #4**	1,547	6.29	53.2
**ZF Facility 3 - Sample #1**	2,008	7.04	391.5
**ZF Facility 3 - Sample #2**	1,794	7.10	401.7
**ZF Facility 3 - Sample #3**	1,984	7.05	395.9
**Mean**	**1,984**	**7.01**	**407**
**SD**	**431**	**0.26**	**147**
**N**	**10**	**10**	**10**
**Min**	**1,468**	**6.29**	**53**
**Max**	**2,826**	**7.20**	**613**

**Table 3.** Water chemistry parameters measured at the university of Exeter at multiple sites (samples 1-3).

Sample description	pCO _2_ (μatm)	pH	Alkalinity (μM)
**Exeter - Sample # 1**	524	7.39	233.5
**Exeter - Sample # 2**	436	7.57	306.1
**Exeter - Sample # 3**	435	7.42	219.0
**Mean**	**465**	**7**	**253**
**SD**	**51**	**0.10**	**47**
**N**	**3**	**3**	**3**

### Experiment 1: Analar grade salts and CO _2_

In this experiment, we exposed zebrafish to 9 different levels of Analar grade salt concentrations (5 to 1000 μM Na ^+^) at 4 different CO _2_ levels (400 to 8000 μatm CO _2_) to investigate how salinity and CO _2_ affect the growth and early development (0-4 dpf) of zebrafish. Fertilisation success of embryos in all treatments was >87%, above the required 70% ( OECD *Test No. 236: FET*, 2013). In all the treatments survival was above the required 80 % ( OECD *Test No. 236: FET*, 2013), and above 91% except for the larvae exposed to the lowest sodium level (5 μM) and 4000 μatm CO _2_ which had 83% survival. Zebrafish developed normally, showing tail detachment, somite formation, hatching success and heartbeat, regardless of salt and CO _2_ treatment (Supplemental material Table S7). Zebrafish larvae exposed to 5 μM Na ^+^ and 4,000 μatm CO _2_ had a reduced response to touch (Supplemental material Table S7). By 4 dpf, ~40% of the larvae had an inflated swim bladder, except those exposed to 5 μM, in which almost none had an inflated swim bladder (Supplemental material Table S7). Swim bladder inflation was also lower in larvae exposed to 2,000, 4,000 and 8,000 μatm CO _2_ compared to those exposed to 400 μatm CO _2_.

Larval length at 4 dpf varied between 3,239 μm and 4,150 μm ( Figure 4) and was significantly affected by both CO _2_ and salt concentration (p<0.0001 for both), but the interaction between CO _2_ and salt concentration was not significant (p=0.071). Generally, at all CO _2_ levels larvae exposed to lower salt concentrations (5 to 100 μM) were significantly smaller by ~5% than those exposed to intermediate and higher concentrations or the system water. Larvae exposed to 2,000 and 4,000 μatm CO _2_ were 2.2 and 2.7% longer than those exposed 400 and 8,000 μatm CO _2_, respectively.

### Experiment 2: Commercial marine salts and CO _2_

As most facilities use commercial salts, we repeated the above experiment using 2 different commonly used marine salts (Instant Ocean and Tropic Marin ^®^) at 6 different salinities (0.08, 0.16, 0.32, 0.64, 1.2 and 2.0 ppt for each one) and 3 different CO _2_ levels. Average fertilisation success was >85%. The average survival was above 95% in most experimental treatments; however, the survival of zebrafish larvae exposed to 4000 μatm CO _2_ was generally lower (only 83%) especially when exposed to lower or higher salt concentrations (0.08, 0.16 and 2.0 ppt). Zebrafish developed normally, showing tail detachment, somite formation, hatching success, heartbeat, and response to touch regardless of salt and CO _2_ treatment (see Supplemental Material Table S8). Similarly to experiment 1, larvae exposed to the lowest salt concentrations (IO 0.08 and TM 0.08) had the lowest rates of swim bladder inflations compared to those larvae exposed to higher salt concentrations (see Supplemental Material Table S8). Swim bladder inflation was also lower in larvae exposed to 400 and 4,000 μatm CO _2_ compared to those exposed to 2,000 μatm CO _2_.

Larval length at 4 dpf varied between 3,267 μm and 4,015 μm ( Figure 5) and was significantly affected by pCO _2_ and salt concentration (p<0.001 for both), but the interaction between pCO _2_ and salt concentration was not significant (p=0.739). Overall, zebrafish larvae exposed to the intermediate CO _2_ level of 2,000 μatm were 1 to 2% longer than those exposed to 400 μatm CO _2_ (control) and 1 to 3.5% longer than those exposed to the highest CO _2_ of 4,000 μatm ( Figure 5). There were significant differences in larval length between the brads of marine salts at 0.16 and 2.0 ppt ( Figure 5; p <0.05), but not at other salt concentrations.

## Discussion

**Figure 3.**
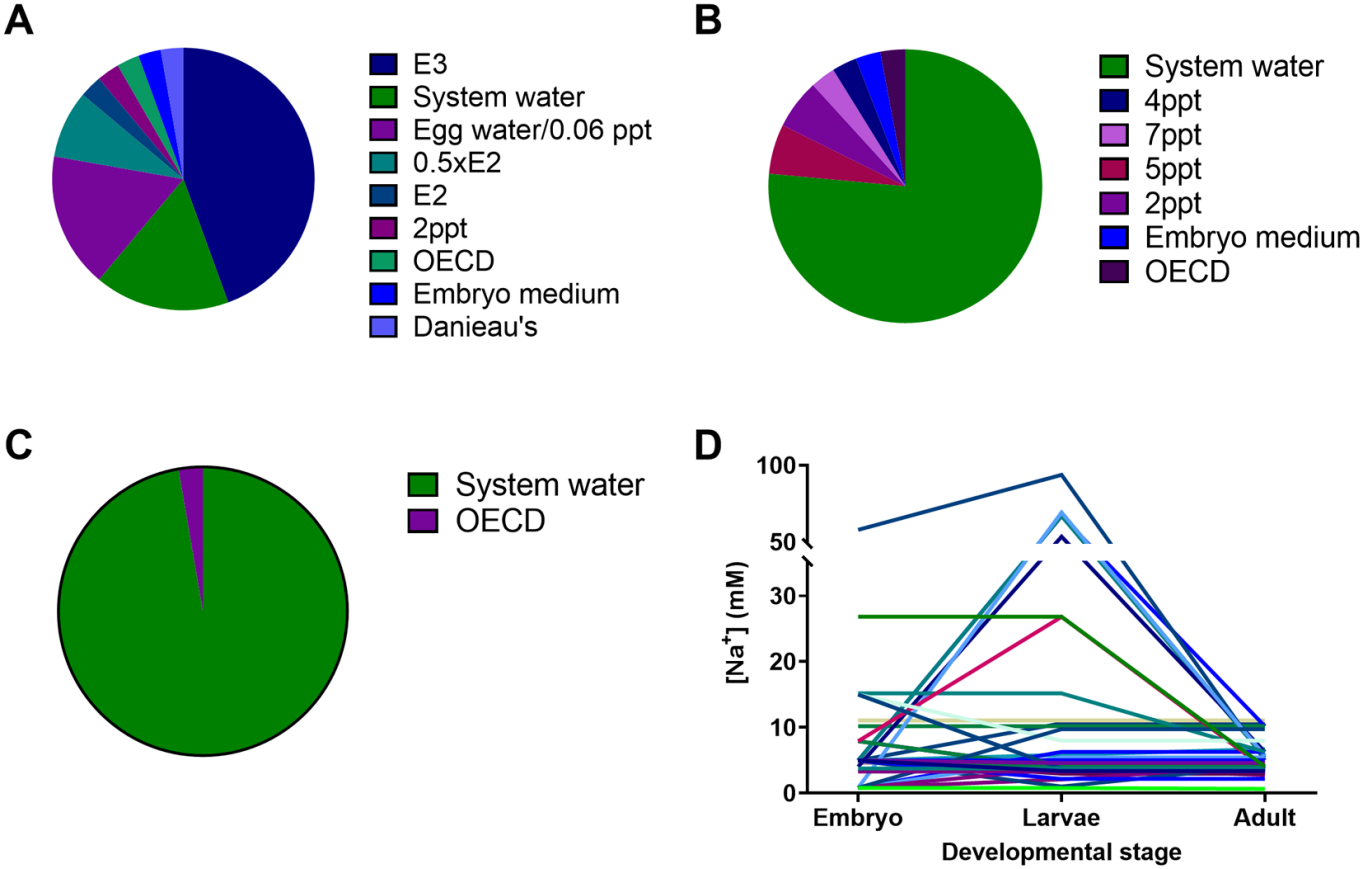
Make up water recipes used to house zebrafish embryos/larvae up to 4-6 dpf (A); larvae from 4-6 and up to 8-21 dpf (B); larvae older than 21 dpf (C) and the range of sodium ion concentrations (mM) (D) in these different solutions that zebrafish experience in the 40 different institutions surveyed. Of note: the system water refers to the standard water used for housing fish in each facility, but the salt concentrations in these systems varied by up to five-fold, therefore not one standardized recipe.

**Figure 4.**
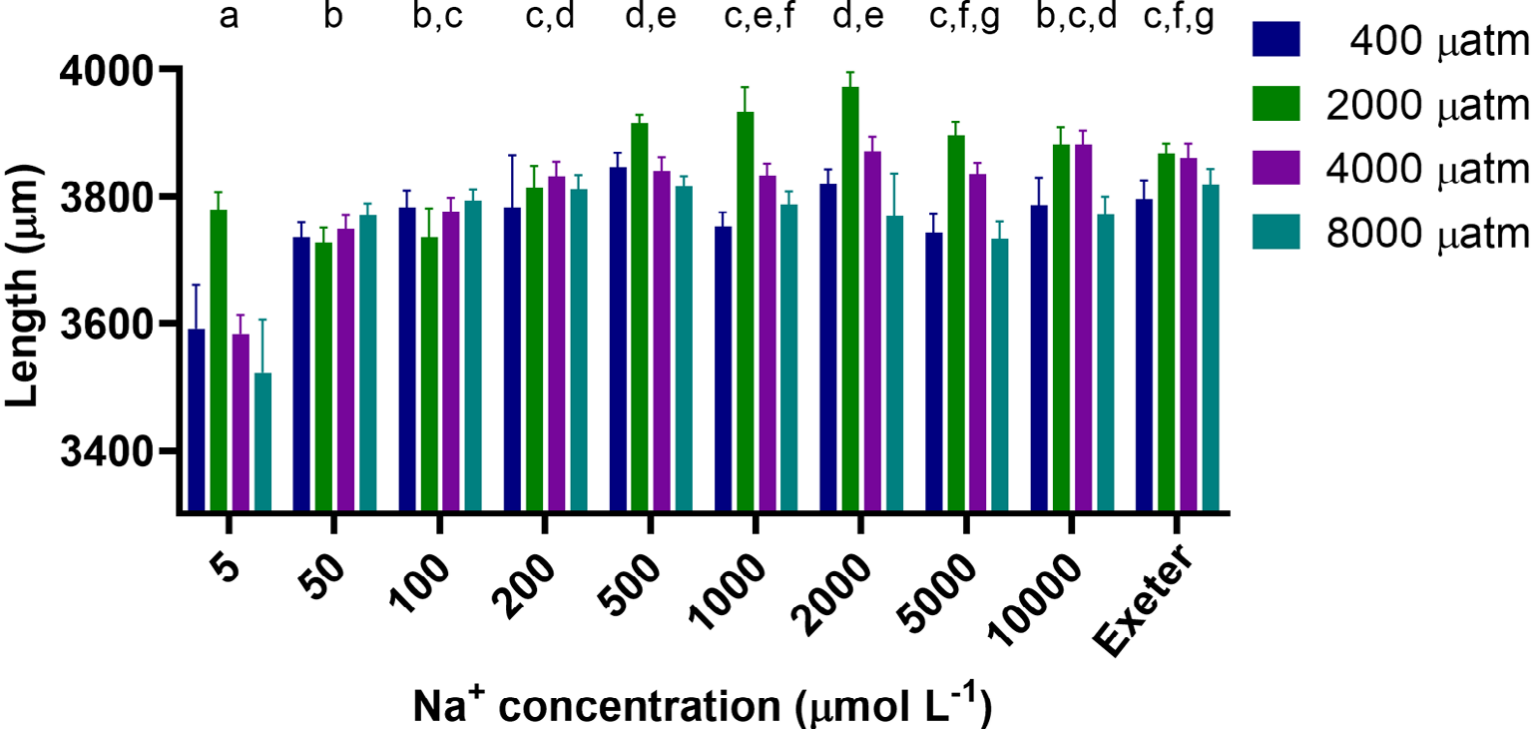
The effect of Analar grade salt and CO _2_ exposure on the body length (μm) of 4 dpf zebrafish larvae (n = 18-24 per treatment, except 5 μM at 2,000 μatm where N = 11). Different lower case letters above a salt concentration indicate significant differences between these salt concentrations (p<0.05), those that share at least one letter are not significantly different than one another; length was significantly different between all pCO _2_ levels (p<0.05). Exeter refers to the system water at the University of Exeter which was used as a reference.

**Figure 5.**
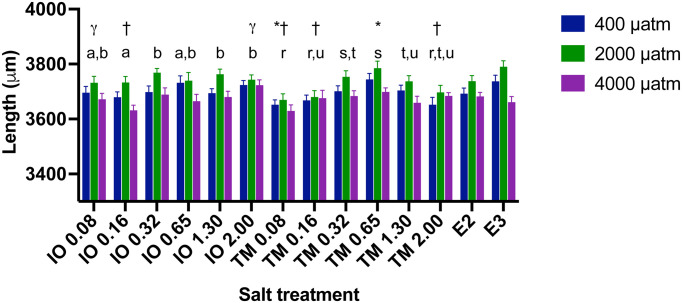
The effect of commercial marine salt and CO _2_ exposure on the body length (μm) of 4 dpf zebrafish larvae (n = 19-24 per treatment). Different lower case letters above a salt concentration indicate significant differences between these salt concentrations within salt treatments (p<0.05), those that share at least one letter are not significantly different than one another; * indicate significant differences from E2 (p<0.05), † indicate significant differences from E3 (p<0.05), and γ indicates significant differences between salt brands at that salinity. Length was significantly different between all pCO _2_ levels (p<0.05). IO indicates Instant ocean and salinity level in ppm, TM indicates Tropic Marine and salinity level in ppm. E2 and E3 refer to the freshwater media used in zebrafish research (see Nüsslein-Volhard and Dahm 2002).

Our survey confirmed that there was considerable variability in the water chemistry used between different institutions ( Figures 1- 3), with ion concentrations varying as much as 122-fold between institutions at certain life stages. In laboratory experiments, we tested a large range of salt concentrations that spanned the entire range encountered in these facilities in combination with 3 to 4 different pCO _2_ concentrations and found that zebrafish larvae exposed to low salt concentrations developed normally, but were smaller than zebrafish exposed to higher salt concentrations ( Figures 4, 5). Additionally, larvae exposed to different pCO _2_ concentrations did not have any developmental abnormalities, but those exposed to intermediate levels of pCO _2_ were the longest suggesting faster growth. Overall, zebrafish larvae were robust in terms of their development and growth when exposed to a large range of salt and CO _2_ levels, but what effects these early exposures may have on later developmental stages needs further investigation.

### Survey results

Most institutions (36) reported using reverse osmosis (RO) water as the basis for their system water, with only four institutions choosing to use dechlorinated water. In all but one institute (that used dechlorinated tap water), salts are then added to reconstitute the water to their desired recipe. The institute using dechlorinated water without additional salts is located in an area with very hard water and presumably contains sufficient salts for the welfare of zebrafish. However, the chemistry of dechlorinated tap water varies enormously with local geology, and even in the same place it varies over time with the seasons/weather and depending on the source reservoir being used and operational changes at the mains water plant. Therefore, its consistency is not guaranteed spatially or temporally with potentially important consequences for experimental outcomes and reproducibility of studies. Using RO water is more costly and results in higher total water usage due to the portion discarded by the RO filtration process. On the other hand, by using RO water reconstituted with added salts, zebrafish facilities are better able to standardise and control their system water conditions. In our survey, during the stages to independent feeding (< 5 dpf) fish were exposed to the most diverse range of freshwater media types across institutions ( Figure 3A). For larvae from 5 to 21 dpf the number of freshwater media types decreased with the system water becoming the dominant choice ( Figure 3B). With the exception of one facility, all larvae post 21 dpf were transferred to system water following this life stage ( Figure 3C). Notably, larvae from 5 to 21 dpf are likely to be exposed to the greatest variation in ion concentrations during this life stage with up to a 87 fold change in Na ^+^ concentration ( Figure 3D). Additionally, many institutions reported switching (from using previously published freshwater media such as E2, E3, embryo water and Danieau’s solution for embryos and larvae) to system water. This switch to system water is likely driven by convenience as well as the ability to achieve a more consistent system water composition over time within a given institution.

Most institutions maintained conductivity, pH and temperatures within the recommended range for zebrafish of 150 to 2,000 μS/cm ( Goodwin *et al.,* 2016; Harper and Lawrence, 2011; Martins *et al.*, 2016), pH of 6.5 to 8 ( Aleström *et al.,* 2020), and 24 to 31°C ( Westerfield, 2007), respectively. However, these ranges are wide, with the H ^+^ concentration varying by 32-fold and conductivity by 13-fold between the lowest and highest recommended values, respectively. These ranges are consistent with the wide variation in water chemistry that zebrafish are adapted to in their natural environment ( Aleström *et al.,* 2020). However, they are still sufficiently large to be biologically significant and so we propose that these should be narrowed across zebrafish facilities to minimize variation in phenotypes, although more research is necessary to confirm the quantitative significance of this suggestion. A striking discovery from the survey was the relatively wide variation in salinity that zebrafish larvae were exposed to in a relatively short period of time (2 weeks) and during critical stages of development. We did not test the effects of these large temporal changes in salinity on growth or development, but this warrants further investigation to determine if such large fluctuations could have effects lasting into the juvenile stage or adulthood. A current study over the whole life cycle aims to determine what water chemistry is optimal based on growth, physiological and behavioural performance indicators. Such studies are needed before guidelines and recommendations can be made for facilities to adopt.

### pCO _2_ levels from large zebrafish facilities

The pCO _2_ in the water samples taken from three major zebrafish facilities varied between 1,468 and 2,826 μatm, much higher both maximum and range than the 450 to 1,200 μatm previously reported at four different biomedical facilities in Sweden ( Vossen *et al.*, 2016). These partial pressures are almost 3 times as high as the pCO _2_ predicted in the atmosphere and surface ocean for year 2100 under a business-as-usual scenario of anthropogenic CO _2_ emissions ( IPCC, 2013). However, pCO _2_ levels vary much more in freshwater environments ( Weiss *et al.*, 2018) due to variations in local geology, water cycle, vegetation, and climate ( McNeil and Matsumoto, 2019). High pCO _2_ values up to 10,000 μatm are common in both freshwater and marine recirculating aquaculture systems (RAS) used for intensive aquaculture ( Ellis *et al.*, 2017). The facilities used for the culturing of zebrafish are very similar in principle (but usually smaller in scale) to those used in large-scale aquatic food production systems because many thousands of fish share the same recirculating water. However, in our experiments zebrafish larvae exposed to 2000 μatm were the longest. These pCO _2_ values are well within those reported for zebrafish in the wild (~4500 μatm) ( Sundin *et al.*, 2019). It is unclear whether the larvae grew best at 2000 μatm because they have been exposed to this level over several generations in a laboratory setting or because they have evolved in a high pCO _2_ environment in the wild, or both. Therefore, ambient levels of pCO _2_ (i.e., the lowest used in our experiments; 400 μatm) often deemed the “norm” and employed as the control condition in climate change studies in various fish species, may not provide optimum growth in zebrafish and perhaps should not be targeted in zebrafish facilities. However, longer duration exposures than just these early life stages will help expand our understanding of optimal CO _2_ levels for zebrafish more generally.

### Effects of salt and pCO _2_ on the development of zebrafish larvae

None of the salt concentrations used in either experiment limited the ability of zebrafish to reach different developmental stages, but potential negative effects on swim bladder inflation were observed at low the lowest salt concentrations when combined with high pCO _2_ of 2000 μatm and above (Experiment 1, Supplementary table S7) or at 400 and 4000 μatm CO _2_ (Experiment 2, Supplementary table S8). As measurements were carried out at 4 dpf it is unclear if any fish would still lack an inflated swim bladder at 5dpf when zebrafish larvae are generally placed in nursery tanks with deeper water. If so, this would increase mortality rates due to inability to reach the surface of the deeper water to inflate their swim bladder or impair swimming. Therefore, careful monitoring of swim bladder inflation might be particularly important for those labs or institutions that use water from their racks to raise zebrafish larvae as it tends to be higher in pCO _2_ than freshly made media (i.e. E2 or E3). Furthermore, zebrafish larvae exposed to some of the lowest salt level in each of experiments 1 and 2 (5 μM Na ^+^ and 0.16 ppt sea salt, respectively) were shorter than those exposed to intermediate salinities (500 to 2,000 μM Na ^+^ in Experiment 1, and 0.32 to 1.3 ppt sea salts in Experiment 2) and similar in size to those exposed to high salinities (1 mM Na ^+^ in Experiment 1, and 2 ppt in Experiment 2), indicating that intermediate salt concentrations provided the optimal growth in zebrafish larvae. Freshwater fish need to actively uptake ions from the external water to maintain ionic homeostasis, and the active transport processes usually follow classic Michealis-Menten kinetics ( Potts, 1994), i.e. a steep rise followed by a plateauing of the active ion uptake rate as the external water ion concentration increases. Thus, zebrafish exposed to low salt concentrations will have impaired capacity for active ion uptake ( Kwong and Perry, 2013; Kwong *et al.,* 2013) and may be compromised in their ability to fully compensate for the constant passive diffusive ion losses, but studies to date have not assessed ion regulation in zebrafish larvae exposed to such low ion concentrations (5 μM Na ^+^). However, it is conceivable that zebrafish larvae could compensate for living in a low salinity environment once feeding independently by obtaining additional salt from their food, as seems to be important in many fish species that live in ion poor waters ( Gonzalez *et al.*, 2005). But this warrants further investigation for zebrafish. Although high salinity of more than 2 ppt could affect zebrafish growth by impairing nuclear division of the embryonic cells during early development ( Sawant *et al.*, 2001), salinity levels of around 2 ppt have been reported to cause the most rapid growth ever reported in zebrafish when combined with constant light conditions and live rotifer feed ( Dabrowski and Miller, 2018).

Zebrafish can detect high CO _2_ levels of >10,000 μatm CO _2_ using neuroepithelial cells in their gills ( Qin *et al.*, 2010) or levels of > 30,000 μatm CO _2_ through the terminal nerve near the olfactory epithelium ( Koide *et al.*, 2018). These high CO _2_ levels induce a slow avoidance behaviour in zebrafish larvae ( Koide *et al.,* 2018), but they are much higher than those we report here for 3 UK research facilities or for zebrafish habitats in the wild ( Sundin *et al.,* 2019). However, a study looking at levels of CO _2_ that more closely resemble those reported here for research facilities found that zebrafish adults exposed to 1600 μatm CO _2_ exhibited a stronger turning preference (lateralization) than those fish exposed to 400 μatm CO _2_ ( Vossen *et al.*, 2016). This is opposite to that found in marine fish which show a reduced turning preference at higher CO _2_ (1000 μatm) compared to ambient CO _2_ (400 μatm) ( Domenici *et al.*, 2012). Preferring to turn one way over another (i.e. being lateralized) is considered to be more normal for fish than not having a preference. These findings therefore may indicate that 1600 μatm CO _2_ is the more common condition for zebrafish (or to what they have adapted to) and that 400 μatm CO _2_ is perhaps the more unusual condition. Furthermore, exposure to 1600 μatm CO _2_ did not affect the activity of adult zebrafish compared to those exposed to 400 μatm CO _2_ ( Vossen *et al.*, 2016), but higher pCO _2_ levels were not tested, and neither were other stages of development.

Therefore, levels of pCO _2_ encountered in research facilities could affect behaviour of zebrafish, as indeed may sudden transfer between waters of different pCO _2_ (as may occur when conducting behavioural assays or drug or toxicant exposures in different water to the home tank). However, further studies are necessary to determine if this is the case, at which stages of development this might be happening and at what pCO _2_ levels, keeping in mind that ambient CO _2_ (~400 μatm) may not necessarily represent the control condition for zebrafish.

Longer term studies in other fish species suggest that low salt levels act as a mild environmental stressor with a higher cost of living indicated by detrimental changes in growth, feeding and protein turnover ( Reid *et al.*, 1995), whilst longer term exposure to elevated CO _2_ can also compromise growth with adverse effects on immune function genes, thinner skin (dermis and epidermis) and fewer mucous cells, with implications for defence against infectious agents ( Mota *et al.*, 2019, 2020). Future work will involve full life-cycle (embryo to adult) exposure of zebrafish to a range of salt and CO _2_ environments such that we can investigate the impacts of these variables on multiple life history stages, and end points that matter the most to zebrafish facilities. End points will include growth rate, fish condition and time to maturation; reproductive success, most notably egg output and gamete quality; physiology of ion regulation and its impact on, for example, acid-base regulation in response to routine husbandry stresses, feed conversion efficiency; and immune function which impacts the health/robustness of the population during to day-to-day husbandry practices and susceptibility to infection in large shared recirculating systems.

## Conclusions

Here we highlight the variability in water chemistry used to house zebrafish for scientific research despite their use since the 1960’s. We show that these variations can lead to changes in growth of embryos up to 4 dpf but whether these changes manifest in later life performance or have the potential to impact the outcomes of research studies needs further investigation. At intermediate levels of CO _2_ (2000 μatm) larvae were significantly longer, but also tended to have lower swim bladder inflation rates when water was made with Analar grade salts. Therefore, higher CO _2_ levels might have both positive and negative effects on zebrafish larvae and particular attention should be paid to swim bladder inflation before zebrafish are transferred to deeper water as this is critical for larval survival and performance. Moreover, CO _2_ levels that are too high (4000 and 8000 µatm) reduced the growth of zebrafish larvae and this is consistent with previous research showing salmon larvae exposed to high CO _2_ had a reduced efficiency of conversion of yolk into growth ( Ou *et al.*, 2015). Additionally, as current guidance is variable and often vague ( Lee *et al.*, 2022), we highlight the lack of information to inform guidance for zebrafish welfare. As there are over 5 million adult zebrafish used for research annually worldwide (many more if larvae younger than 4 dpf are considered), optimizing and standardizing water chemistry parameters presents an opportunity to improve zebrafish welfare. This would be through refinement due to improved fish health. Narrower ranges of water chemistry would reduce the number of fish growing in suboptimal conditions (too low or too high salinity, CO _2_, pH or temperature) as these fish would be investing more energy into maintaining homeostasis and, therefore, have less energy available for growth, development or to mount an immune response. Additionally, this variability in energy allocation could lead to increased variability in responses during experimental measurements increasing the variability, sample size and use of zebrafish for research purposes. Therefore, a reduction of just 5% of the zebrafish used in research due to more optimal husbandry water chemistry conditions would represent over 250,000 fewer fish being used as a consequence of decreased variability of results and improved replicability. Therefore, we propose that a better understanding of what drives facility choices in the water chemistry ranges they adopt is needed. We propose that these choices are aligned better with peer-reviewed scientific evidence if we are to optimise and standardise approaches between institutions, develop best practice for fish welfare, improve performance and reduce the potential causes of reproducibility problems in research using zebrafish. Institutions are currently measuring several important water chemistry parameters, but these are often not reported (or not reported accurately) in the methods section of scientific outputs. In addition to the information minimum recommended to be included in a manuscript under the ARRIVE guidelines, we recommend that for aquatic animals researchers should report temperature, pH, conductivity and the nominal recipe for the freshwater media used for their stock as well as experimental animals used, and ideally this should be confirmed by direct measurement of the biologically important inorganic ions if possible (especially sodium, chloride, calcium) as well as alkalinity.

### Ethical considerations

All experiments were carried out in the University of Exeter Aquatic Resources Centre (ARC), and procedures were approved by the Home Office (License No P88687E07) and reviewed by the University of Exeter Ethics (Application ID 5542334). All relevant information has been reported in the body of the manuscript in line with the ARRIVE 2.0 guidelines developed by the NC3Rs, including our efforts to ameliorate any suffering of animals.

## Data Availability

The underlying data has been deposited in Figshare: https://doi.org/10.6084/m9.figshare.22644607.v1 ( Porteus *et al.*, 2023). This project contains the following underlying data:
• Porteus et al F1000 all raw data.xlsx (Data file 1 includes all the raw data from the survey responses and Experiments 1 and 2) • Supplementary materials file.pdf (Supplementary materials file) • ARRIVE Author checklist.pdf (ARRIVE guidelines) The results of the statistical analyses are deposited in Figshare:
•
https://doi.org/10.6084/m9.figshare.26166814 Data are available under the terms of the Creative Commons Attribution 4.0 International license (CC-BY 4.0).
