# Supplementary material for: A survey of water chemistry used in zebrafish facilities and their effects on early zebrafish development
Source: F1000Res. 2024 Aug 20;13:168. Originally published 2024 Mar 8. [Version 2] doi: 10.12688/f1000research.134520.2 (PMC11462130; doi:10.12688/f1000research.134520.2)
Supplement: Peer review history

# Reviewer report: Reviewer response for version 2

**DOI:** 10.5256/f1000research.170386.r329005

**Published:** 2024-10-24

**Authors:** Christopher Pierret

**Affiliations:** 1 Mayo Clinic Minnesota, Rochester, Minnesota, USA

This article on water chemistry and zebrafish development explores the wide variety of approaches that are being used to rear zebrafish for research at facilities around the world. The article appears to have been initially reviewed and has come back in an improved form. Many of these data are truly interesting. I am left with one significant concern with regard to the experimental design in the water chemistry comparison and another moderate concern about the conclusions drawn. 

 The authors appropriately note that not only is the conductance varied across facilities, but so are the CO2 levels. Further there is a description of variability in the media used to raise the embryos in each facility. 

 My first concern: Left unaddressed is the variability in zebrafish strains used in these same facilities. The experiment uses only one strain of zebrafish and uses a transition from system water to the variety of testing conditions. Certainly one could imagine that as testing conditions vary more significantly from the system water at the University of Exeter, the more dramatic the developmental differences. This information cannot be generalized across facilities, as each has different strains of zebrafish in differing system water levels, then moved to facility choice of embryo medium. To properly determine a narrower range of water chemistry, one would want to start with adults representing different strains in a variety of conditions, then raise clutches of embryos in system water versus other media. This would assure that the results were reflective of the conditions overall.  A second experiment would then be set up to show the effect of embryo media versus system water.

 Without this change to the approach, the experiment is only determining this strain in this system condition's response to changing water chemistry conditions during embryonic development. It does not result in data that can inform a more standard approach. Therein lies my next (moderate) concern. The authors choose to close the article without proposing an improved approach. If the experiment does not allow the authors to present a better approach, I'm not sure what the value of the article would be to readers. 

 I am aware that follow up experiments from reviewers can be misaligned with time available of the authors. I am stuck, however, finding value in the indexing of this work without them.

Is the work clearly and accurately presented and does it cite the current literature?

Yes

If applicable, is the statistical analysis and its interpretation appropriate?

Not applicable

Are all the source data underlying the results available to ensure full reproducibility?

Yes

Is the study design appropriate and is the work technically sound?

Partly

Are the conclusions drawn adequately supported by the results?

Partly

Are sufficient details of methods and analysis provided to allow replication by others?

Yes

Reviewer Expertise:

Biochemistry and Molecular Biology

I confirm that I have read this submission and believe that I have an appropriate level of expertise to state that I do not consider it to be of an acceptable scientific standard, for reasons outlined above.

# Reviewer report: Reviewer response for version 2

**DOI:** 10.5256/f1000research.170386.r329012

**Published:** 2024-10-08

**Authors:** Ziyu Guo

**Affiliations:** 1 Guizhou Medical University, Guiyang, China

The manuscript "A survey of water chemistry used in zebrafish facilities and their effects on early zebrafish" confirms that there was considerable variability in the water chemistry used across different institutions, despite these institutions all followed certain standard methods. Further experimental results on animals indicate that short-term exposure of zebrafish embryos to varying ranges of salt concentrations and pCO _2_ did not result in noticeable developmental or growth damage. However, differences in indicators such as swim bladder formation and body length still suggest this is an issue that cannot be ignored. This study provides a valuable contribution to understanding experimental results and reproducibility in the field of zebrafish research (also prompting deeper reflections into my own future research), but there are some points that need improved. The suggestions for improvement are as follows:

1. Abstract - There are some fundamental formatting errors that should not be present.       
2. Abstract, Methods - The logic is not clear enough. Consider moving some content to the Results section, and there is a lack of presentation of the observed indicators and exposure periods in the zebrafish experiments.
3. Abstract, Results - Further improvements are needed, as the current version is quite rudimentary. The survey section is also an important result of this study and should not be overlooked.
4. The selection of keywords needs to be concise, and in order to improve retrievability, words that already appear in the title should be appropriately avoided.
5. It is suggested to remove redundant statements in the title of Table 1.
6. The introduction is well written, but the logical expression of the last paragraph still needs to be optimized, and it can provide a more detailed introduction to the work carried out in this study.
7. Methods - No introduction was found on the method of estimating Na ^+^ concentration based on conductivity. It is suggested to supplement this.
8. Methods - The specific statistical methods used for data analysis need to be specified. It may be necessary to consider variations in the choice of statistical methods due to differences in sample sizes among treatment groups, if this has not been accounted for in previous analyses.
9. It is suggested to add more specific data to the pie charts in Figure 1 and Figure 3.
10. The investigation found differences in the set-points and acceptable ranges of temperature, pH, and conductivity in the zebrafish culture water among various institutions worldwide. Why were no further animal experiments conducted on temperature, given that it is also emphasized in the abstract and results? Should this be considered for inclusion in the discussion, if relevant content has already been reported?
11. The black horizontal lines in Figure 2a, b, and c are confusing in terms of what they represent.
12. The survey results indicate that 14 dpf zebrafish larvae in an institution experience a 20.5-fold change in sodium concentration within approximately 24 hours. This finding is interesting; has there been no further investigation into its potential implications?
13. A suggestion is not to present such imprecise data in the results: ~40% of the larvae.      
14. Is the text description of Figure 3 not included in the results?
15. It is suggested that the authors check the scientific and clear presentation of the letter symbols used in the statistical analysis results in Figure 4. I cannot clearly discern from the figure whether there is a significant effect between sodium concentration and larval length, nor can I determine the interaction between pCO _2_ and sodium concentration. Additionally, the current method of using letters to indicate significance may also have issues; why does 'a' appear in the treatment group with the lowest larval length instead of the highest? Figure 5 also needs to be checked for potential problems.

Is the work clearly and accurately presented and does it cite the current literature?

Partly

If applicable, is the statistical analysis and its interpretation appropriate?

Partly

Are all the source data underlying the results available to ensure full reproducibility?

Yes

Is the study design appropriate and is the work technically sound?

Yes

Are the conclusions drawn adequately supported by the results?

Partly

Are sufficient details of methods and analysis provided to allow replication by others?

Yes

Reviewer Expertise:

Ecotoxicology;  Environmental monitoring and evaluation

I confirm that I have read this submission and believe that I have an appropriate level of expertise to confirm that it is of an acceptable scientific standard, however I have significant reservations, as outlined above.

# Reviewer report: Reviewer response for version 2

**DOI:** 10.5256/f1000research.170386.r315482

**Published:** 2024-08-23

**Authors:** Alex Zimmer

**Affiliations:** 1 University of New Brunswick Saint John, Saint John, New Brunswick, Canada

The authors have sufficiently addressed all of the comments that were raised in my initial review. I have no further comments.

Are the 3Rs implications of the work described accurately?

Yes

Is the work clearly and accurately presented and does it cite the current literature?

Yes

If applicable, is the statistical analysis and its interpretation appropriate?

Partly

Are all the source data underlying the results available to ensure full reproducibility?

No source data required

Is the study design appropriate and is the work technically sound?

Yes

Are the conclusions drawn adequately supported by the results?

Partly

Are a suitable application and appropriate end-users identified?

Yes

Are sufficient details of methods and analysis provided to allow replication by others?

Yes

Reviewer Expertise:

Fish physiology; Developmental physiology; ionoregulatory physiology

I confirm that I have read this submission and believe that I have an appropriate level of expertise to confirm that it is of an acceptable scientific standard.

# Reviewer report: Reviewer response for version 1

**DOI:** 10.5256/f1000research.147581.r264116

**Published:** 2024-05-03

**Authors:** Alex Zimmer

**Affiliations:** 1 University of New Brunswick Saint John, Saint John, New Brunswick, Canada

Comments on Porteus et al.

                Porteus et al. present a study that describes the range of water chemistry conditions that are currently used in zebrafish facilities of many major research institutions across the globe. They highlight the large discrepancy of water chemistry conditions used across these institutions pointing to potential issues with repeatability of studies, which could result in increased unnecessary use of research animals. The authors go on to conduct a study to test this idea wherein zebrafish embryos were reared under differing concentrations of salts and/or CO _2_ partial pressures that reflect the range used across institutes reported in their survey. In general, their results demonstrate that fish reared under extremely low levels of salts (5 µM Na) showed reduced growth relative to the other concentrations. While other subtle differences were also apparent, these were not as striking as the effects at the lowest salt concentration. Overall, I found this study to be thoughtfully designed and well-implemented. Moreover, I feel that this study will be a useful reference for researchers in the zebrafish community looking to optimize their culture conditions. Indeed, the authors point out many pitfalls (e.g., large changes in salt concentration between life stages) that could be avoided with more careful control of rearing environment. My major criticism with the manuscript in its current form is that I feel that some of the conclusions, for example that all research institutes need to improve control and standardization of water chemistry conditions, are not supported by the data presented. As I discuss in more detail below, I feel like the results could be painted in a more positive light than they are in the current form.

 Specific comments:

1. Small point (line 3, paragraph 1 in the Introduction), but is it worth mentioning calcium as another important ion that fish must take up from the water? Certainly, this must have important implications for fish growth.
2. Figure 4. The statistical notations in this figure are very hard to decipher as presented. For instance, from my interpretation it seems like the 500 uM Na treatment (noted with “d,e”) is statistically different from the Exeter treatment (noted with “c,f,g”), but this strikes me as hard to believe because some of the CO _2_ treatments are higher in the 500 uM treatment (400 and 2000 uatm), some are lower in the 500 uM treatment (4000 uatm), and another is more-or-less equal (8000 uatm). I suggest double-checking the notations in this figure for accuracy. Apologies if I have misinterpreted the notations.
3. The authors conclude generally that optimizing and standardizing water chemistry parameters present an opportunity to improve zebrafish welfare, potentially reducing worldwide usage by 250,000 fish per year if changes are sufficient to reduce usage by 5%. While I agree that reducing animal usage in research is important, I feel that the data the authors present suggest that zebrafish are quite hardy and seem to perform well across a wide range of water chemistry conditions (e.g., growth is unchanged across ion concentrations ranging 2-3 orders of magnitude!). Clearly more work is needed to understand whether these differences have knock-on effects later on in development or if they alter immune function or other important welfare parameters. However, in my opinion the data represent a “good news” story: despite large differences in water chemistry used across research institutions, zebrafish growth performance is generally consistent across rearing conditions. The one real difference in the study was observed at 5 uM Na which I imagine would actually be quite hard to achieve in recirculating systems. Therefore, I suggest that the authors might reconsider their conclusion in light of the fact that their findings appear to paint a more optimistic picture regarding the variability of data produced by different research institutes, at least in the context of early life growth performance.

Are the 3Rs implications of the work described accurately?

Yes

Is the work clearly and accurately presented and does it cite the current literature?

Yes

If applicable, is the statistical analysis and its interpretation appropriate?

Partly

Are all the source data underlying the results available to ensure full reproducibility?

No source data required

Is the study design appropriate and is the work technically sound?

Yes

Are the conclusions drawn adequately supported by the results?

Partly

Are a suitable application and appropriate end-users identified?

Yes

Are sufficient details of methods and analysis provided to allow replication by others?

Yes

Reviewer Expertise:

Fish physiology; Developmental physiology; ionoregulatory physiology

I confirm that I have read this submission and believe that I have an appropriate level of expertise to confirm that it is of an acceptable scientific standard, however I have significant reservations, as outlined above.

# Response

**Published:** 2024-08-13

**Authors:** Cosima Porteus

**Affiliations:** Biological Sciences, University of Toronto, Toronto, Ontario, Canada

**1 – The reviewer is correct that calcium is taken up via the gills from water. However, quantitatively this is less important for freshwater fish than gut uptake of calcium (via the diet). Also, the rate of calcium uptake via the gills is substantially less than that of sodium or chloride uptake via the gills, this was why we focused on sodium in this sentence.**

**2 – We have double-checked the statistical analysis and we have found a couple of errors in this figure and the figure has now been corrected. The 500** **µ** **M salt concentration was significantly different, although only just (p=0.047) than the Exeter water. We have also added our statistical analysis results as separate files to Figshare (see Data availability statement).**

**3 – We agree with the reviewer that this is a good news story, however, we only measured growth and scored deformities on the larvae, which are quite severe endpoints. Our effects on the growth of larvae show that there are sublethal effects and we caution against using very low salt concentrations as these might have longer lasting effects. However, as the last sentence mentions, our growth data are for up to 4 dpf only, i.e. early stages only, and before exogenously feeding, and so we need more data on what happens next with growth. We are currently running these experiments and hope to report these soon in a separate manuscript, but wanted to report our initial results here.  We agree that a sodium level as low as 5 µM will be hard to achieve in a recirc system. However, we can confirm that this was the level predicted based on the salinity reported in a published paper and we contacted the authors who confirmed this was not a typographical error in the paper, therefore we decided to test this level because it was the lowest we could find reported in the literature.**

# Reviewer report: Reviewer response for version 1

**DOI:** 10.5256/f1000research.147581.r264114

**Published:** 2024-04-30

**Authors:** Lynne Sneddon

**Affiliations:** 1 Department of Biological & Environmental Sciences, University of Gothenburg, Gothenburg, Sweden

This very interesting article reports on a hot topic in zebrafish husbandry and concerns standardisation of water quality. It is well known that there are a range of water quality parameters that zebrafish can tolerate and thrive in but there are great disparities between facilities due to care staff employing the most effective means of keeping the fish. Typically, facilities use what works and what they can afford. However, this provides cause for concern since this may affect reproducibility or replicability between laboratories. This article seeks to highlight the variable approaches across institutions and then focuses on NaCl and CO2 as factors that may affect development of zebrafish up to 4 days post fertilisation. First a survey is conducted which provides insight into the variability of water quality parameters that facilities are adopting in zebrafish husbandry. Secondly, three institutions are sampled for CO2 analysis and finally an experiment is conducted using high grade salts versus commercially available marine salts combined with differing CO2 levels. The experiments demonstrate that low NaCl using high grade salts affects length and in both salt formulations swim bladder inflation is impaired. Slightly elevated CO2 (2000 atm) appears to increase length at some CO2 concentrations. CO" at 4000 atm has a negative impact on length. In the three institutions sampled, they range from ~1500 to 2800 - I wonder if this is due to the use of sodium bicarbonate that is largely employed in commercially systems to maintain pH - can the authors comment on the fact that this can elevate CO2 above ambient (400 atm)? These seem high to me and would suggest then the life support systems are inadequate for dealing with CO2 - can you please suggest the use of additional aeration or degassing systems in the discussion - get rid of the CO2 = get rid of the problem? Or do you think based on your 4 dpf results these higher than ambient CO2 concentrations are beneficial? Of course many facilities do not keep larval fish on their main systems so as it is you can only suggest these CO2 levels up to 4 dpf. These could have negative consequences on adults but then they are coming from probably large, successful facilities. One of the key issues that should be mentioned possibly in the introduction is that zebrafish are largely used for biomedical studies where the researchers want the animals to be kept at standard or constant environmental conditions - there is an emphasis on environmental studies and toxicology in the article but I think this is a good selling point that for biomedical experiments we need agreed water quality parameters between labs. Can you add statistical analysis to compare between the salts - perhaps it is the way it is written but these are treated like two different experiments and yet really we need to know how they compare. Are we doing the right thing for the fish by using commercially available marine salts or should we use the more precise methods of high grade salts? When looking at the two figures on length it would seem only the low concentrations of high grade salts affect growth and actually this would convince me marine salts are possibly more beneficial. Further was there any difference between salts - it seems from the graph that IO may be better than TM? Overall I think this is a very useful article that can promote standardisation across facilites and highlights the impact of water quality on growth in 4 dpf as well as other factors such as swim bladder inflation. This could help facilities trouble shoot high mortality rates in larvae and provide an avenue of investigation for the improved survival of larval zebrafish. I have some very minor comments and suggestions:

 Abstract, Background - delete importantly as important used in previous sentence.

 Abstract, methods: Add "water chemistry parameters"

 Throughout the manuscript you should add mg/l for CO2 and NaCl concentrations as this is what they are measured in by most water quality probes.

 Abstract, Results: Change to "swim bladders did not"

**Introduction Line 1:** change ion levels to dissolved ion concentrations

 End of paragraph 1: regained would be a better word than recovered and I am not sure what "more completely" means here so I suggest delete. "restoring" - would maintaining be better and was this an experiment so can you add the citation?

 Paragraph 2 -there is a statement on CO2 accumulation - this needs citations

 You use the word cope but just because an animal can cope or tolerate does not mean they are in good welfare? Ca you please add a statement somewhere about this - yes zebrafish can tolerate or cope with a range - it does not mean they are in a good physiological state.

 I would add a sentence to the end of the 2nd paragraph along the lines of "The impact of CO2 or its interaction with NaCl is not clearly understood due to a lack of studies on zebrafish"

 Table 1 - can you provide conductivities? Again mM is not that useful for measuring using commercial aquarium probes - can mg/l be added?

 Survey - I can't seem to access the SI but if not there it would be really useful to have the survey in this section so it could be repeated.

 Water samples - does freezing affect these variables you measured?

 Humane endpoints - would be useful to detail the average % of larvae that reached this point. 

 Results page 7 - can you delete dramatic as it's rather emotive. Discussion, L2 should be laboratory

 P12 typo "the culturing OF zebrafish"

 Delete but perhaps not surprisingly

 Change "measurements were done" to measurements were carried out

 P13 L1-4 you didn't measure lateralisation or preference for turning - it's not mentioned previously so I suggest delete. 

 P13 2nd paragraph delete Therefore as its a new paragraph - alternatively merge with previous.

 Conclusions: CO2 levels that are too high - please give a value to this.

 Mention the ARRIVE guidelines that encourage full reporting of results.

Is the work clearly and accurately presented and does it cite the current literature?

Partly

If applicable, is the statistical analysis and its interpretation appropriate?

Yes

Are all the source data underlying the results available to ensure full reproducibility?

Yes

Is the study design appropriate and is the work technically sound?

Yes

Are the conclusions drawn adequately supported by the results?

Partly

Are sufficient details of methods and analysis provided to allow replication by others?

Yes

Reviewer Expertise:

Zebrafish welfare, Fish behaviour and physiology

I confirm that I have read this submission and believe that I have an appropriate level of expertise to confirm that it is of an acceptable scientific standard, however I have significant reservations, as outlined above.

# Response

**Published:** 2024-08-13

**Authors:** Cosima Porteus

**Affiliations:** Biological Sciences, University of Toronto, Toronto, Ontario, Canada

“I wonder if this is due to the use of sodium bicarbonate that is largely employed in commercially systems to maintain pH - can the authors comment on the fact that this can elevate CO2 above ambient (400 atm)?”

**Response:** **The addition of sodium bicarbonate in recirc systems is typically to modify pH to compensate for the acidification as CO2 accumulates. You are right that whilst this brings pH back up somewhat, it can actually increase the water pCO2 as well, as some of the bicarbonate is converted to CO2 by the excess H+ ions in the water. However, if degassing is sufficient, water pCO2 should return to close to atmospheric levels (400 uatm), but clearly this is not happening in the zebrafish facilities we sampled. In some large scale RAS (e.g. salmon, trout) they use other sources of alkali (e.g. NaOH) which does not add more CO2 whilst buffering pH. However, using caustic soda in this way presents health and safety issues for the staff, so it is a trade off. We do not feel this is information that needs to be added to the manuscript but we offer it here in response to the reviewer.**

 “These seem high to me and would suggest then the life support systems are inadequate for dealing with CO2 - can you please suggest the use of additional aeration or degassing systems in the discussion - get rid of the CO2 = get rid of the problem? Or do you think based on your 4 dpf results these higher than ambient CO2 concentrations are beneficial?”

**Response:** **These CO _2_ levels found in zebrafish facilities are not actually high relative to what we find in commercial aquaculture for food species (e.g. salmon, trout) where 2,000** **µ** **atm is the lowest we have seen, and typically it is closer to 5,000 to 10,000** **µ** **atm as a typical range. However, better degassing would certainly enable lower pCO _2_ values in any system, zebrafish included. As the reviewer comments, we do not yet know all the evidence regarding what the most optimal CO _2_ level is for zebrafish, so we should probably wait until such data are available before making specific recommendations about adding more degassing etc.**

 “Can you add statistical analysis to compare between the salts - perhaps it is the way it is written but these are treated like two different experiments and yet really we need to know how they compare. Are we doing the right thing for the fish by using commercially available marine salts or should we use the more precise methods of high grade salts? When looking at the two figures on length it would seem only the low concentrations of high grade salts affect growth and actually this would convince me marine salts are possibly more beneficial.”

**Response: The reviewer makes a good point that to truly compare these we would need to compare them statistically. However, the experiments were done separately and if any statistical differences were found these could be due to slight differences in ambient temperature, the ratio of the different salts, or differences in embryo batches/parents and, therefore, these analyses were not run. Overall, the embryos seem to grow well in both salt treatments and we don’t see any benefits to using high-grade salts, and we recommend the marine salts as they are more cost-effective and convenient to use than the high-grade salts.**

 “Further was there any difference between salts - it seems from the graph that IO may be better than TM?”

**Response: We initially ran a nested ANOVA with salinity nested within brand that revealed there were no statistical difference between brands. However, we were not able to compare with the two control solutions (E2 and E3) in this analysis. Therefore, we used a two-way ANOVA with salt treatment and PCO2 as factors to allow us to perform an all-pairwise comparison including the control solutions. This analysis indicated significant differences at the lowest and highest salinity (0.08 and 2.0 ppt), and we are indicating this in Figure 5. However, since the middle salt concentrations were not different and the nested ANOVA indicated no significant difference, we concluded that differences between salt brands are negligible. We have also uploaded the statistical results from both analyses as files in a separate Figshare submission.**

 Abstract, Background - delete importantly as important used in previous sentence.

**Response: changed to “Notably”.**

 Abstract, methods: Add "water chemistry parameters"

**Response: Changed.**

 Throughout the manuscript you should add mg/l for CO2 and NaCl concentrations as this is what they are measured in by most water quality probes.

**Response: We have added two new rows to our Table 1 that shows the conductivity in** **µ** **S/cm for each solution (this measures a substance’s ability to conduct electricity and cannot be broken down by individual ion) and one row to show the salinity in ppt. This is what most probes measure. We have also added values in mg/L in the methods and abstract to make it easier to compare pCO2 levels.**

 Abstract, Results: Change to "swim bladders did not"

**Response: Changed.**

 Introduction Line 1: change ion levels to dissolved ion concentrations

**Response: Changed.**

 End of paragraph 1: regained would be a better word than recovered and I am not sure what "more completely" means here so I suggest delete. "restoring" - would maintaining be better and was this an experiment so can you add the citation?

**Response: We have removed “more completely” from this sentence and citations have been added. We simply meant that fish recovered faster to an acid-base disturbance such as elevated CO2 when the water they were in had higher ion concentrations.**

 Paragraph 2 -there is a statement on CO2 accumulation - this needs citations

**Response: Citation added.**

 You use the word cope but just because an animal can cope or tolerate does not mean they are in good welfare? Ca you please add a statement somewhere about this - yes zebrafish can tolerate or cope with a range - it does not mean they are in a good physiological state.

**Response: We have changed the sentence to read “However, just because zebrafish can tolerate these pCO _2_s, they might not be in a good physiological state and thus, despite the ability to regulate blood acid-base balance, living in high CO _2_ water has further, more holistic impacts on fish performance.”**

 I would add a sentence to the end of the 2nd paragraph along the lines of "The impact of CO2 or its interaction with NaCl is not clearly understood due to a lack of studies on zebrafish"

**Response: Thank you. We have added “However, the are no studies on zebrafish that have assessed the impact of pCO2 or its interaction with NaCl.”**

 Table 1 - can you provide conductivities? Again mM is not that useful for measuring using commercial aquarium probes - can mg/l be added?

**Response: Yes, added.**

 Survey - I can't seem to access the SI but if not there it would be really useful to have the survey in this section so it could be repeated.

**Response: The survey is included in the SI. Not sure why the reviewer is unable to access this file.**

 Water samples - does freezing affect these variables you measured?

**Response: The water samples were not frozen, but stored in the fridge (4°C).**

 Humane endpoints - would be useful to detail the average % of larvae that reached this point. 

**Response: Very few larvae, ~0.5% reached the humane endpoints. We have added this information to the manuscript.**

 Results page 7 - can you delete dramatic as it's rather emotive.

**Response: Removed.**

 Discussion, L2 should be laboratory

**Response: Changed.**

 P12 typo "the culturing OF zebrafish"

**Response: Thank you. “of” added.**

 Delete but perhaps not surprisingly

**Response: Deleted.**

 Change "measurements were done" to measurements were carried out

**Response: Changed.**

 P13 L1-4 you didn't measure lateralisation or preference for turning - it's not mentioned previously so I suggest delete. 

**Response: We argue that this is still a valid point even if we did not measure lateralization, as it shows that zebrafish might be adapted to living in higher pCO2 they show a turning preference at 1600** **µ** **atm and not at 400** **µ** **atm.**

 P13 2nd paragraph delete Therefore as its a new paragraph - alternatively merge with previous.

**Response: Merged with previous.**

 Conclusions: CO2 levels that are too high - please give a value to this.

**Response: 4000 and 8000** **µ** **atm have been added in parentheses.**

 Mention the ARRIVE guidelines that encourage full reporting of results.

**Response: We now refer to the ARRIVE guidelines in our conclusion.**
